# Microglial Plasticity in Vascular Dementia: Mechanisms and Therapeutic Reprogramming

**DOI:** 10.3390/ijms27093719

**Published:** 2026-04-22

**Authors:** Manish Shukla, Jarvis Li, Yan Sun, Rong Jin, Guohong Li

**Affiliations:** 1 Department of Neurosurgery, Pennsylvania State University College of Medicine, 500 University Drive, Hershey, PA 17033, USA; manishukladrdo@gmail.com (M.S.); jarvisli@pennstatehealth.psu.edu (J.L.); 2 Department of Cardiology, Jiangsu Province Hospital of Integrated Chinese and Western Medicine, Affiliated Hospital of Nanjing University of Chinese Medicine, Nanjing 210028, China; doctoryansun@yahoo.com; 3 Department of Neurosurgery, Penn State Neuroscience Institute, Pennsylvania State University College of Medicine, 500 University Drive, Hershey, PA 17033, USA; rjin@pennstatehealth.psu.edu

**Keywords:** vascular dementia, microglia, neuroinflammation, white matter injury, synaptic pruning, microglial reprogramming

## Abstract

Vascular dementia (VaD) is a leading cause of cognitive decline and arises from heterogeneous cerebrovascular pathologies, most commonly cerebral small vessel disease and chronic cerebral hypoperfusion. Microglia, the brain’s resident immune cells, exert a dual, stage-dependent influence during VaD progression, initially supporting neuroprotection through debris clearance and tissue repair, but later contributing to chronic neuroinflammation, synaptic loss, and white matter injury. Emerging evidence suggests that multiple molecular pathways, including purinergic receptors, Toll-like receptors and inflammasome cascades, complement-mediated synaptic pruning, and homeostatic and metabolic regulators, such as TREM2 (triggering receptor expressed on myeloid cells 2) and CSF1R (colony-stimulating factor 1 receptor), govern microglial functional transitions. Furthermore, post-transcriptional regulation by microRNAs (e.g., miR-30 family, miR-124, miR-146a, and miR-155) modulates these phenotypes, offering potential biomarkers and therapeutic targets. Understanding these interconnected molecular and epigenetic networks provides a framework for reprogramming microglia from pro-inflammatory to reparative states, thereby providing a mechanistic basis for precision interventions to preserve neurovascular integrity and mitigate cognitive impairment in VaD.

## 1. Introduction

Dementia represents one of the most pressing global public health challenges, with prevalence projected to increase substantially as populations age [1]. VaD is widely recognized as the second most common cause of dementia worldwide, following Alzheimer’s disease (AD). It arises primarily from cSVD, CCH, and recurrent ischemic insults, which frequently coexist and interact pathophysiologically [2].

cSVD is the most common pathological substrate of VaD and is driven by risk factors such as hypertension, diabetes, and aging. These factors promote arteriolosclerosis, lipohyalinosis, endothelial dysfunction, and blood–brain barrier (BBB) breakdown in small penetrating vessels. Such changes lead to lacunar infarctions, microinfarcts, and diffuse white matter injury [3]. These events cumulatively damage the brain by producing repeated thromboembolic or lacunar lesions that disrupt cognitive networks. In parallel, CCH, often secondary to large carotid artery stenosis, cardiac dysfunction, or impaired cerebral autoregulation, reduces cerebral blood flow and promotes white matter lesions, myelin loss, and progressive cognitive decline through sustained hemodynamic insufficiency and associated microvascular pathology [4]. Importantly, in older adults, VaD frequently coexists with AD pathology, resulting in mixed dementia—a multifactorial condition that complicates both diagnosis and therapeutic intervention [5].

Accumulating evidence indicates that cognitive decline in VaD is not merely a consequence of neuronal loss but reflects systemic dysfunction within the neurovascular unit (NVU). The NVU is an integrated multicellular complex composed of endothelial cells, pericytes, vascular smooth muscle cells, astrocytes, and neurons that collectively regulate cerebral blood flow, neurovascular and metabolic coupling, and BBB integrity [6]. Resident microglia are now recognized as functional components of the NVU, particularly in regulating immune homeostasis during neuroinflammation and CNS (central nervous system) disorders [7]. Disruption of NVU function results in inefficient perfusion, impaired clearance of metabolic waste, BBB breakdown, and chronic neuroinflammation, thereby accelerating neural injury and cognitive decline [8,9].

Microglia, the resident innate immune cells of the CNS, are pivotal regulators of neurovascular integrity and responses to cerebrovascular insults. Originating from embryonic yolk-sac progenitors and maintained through lifelong self-renewal, microglia continuously survey the brain parenchyma with highly motile processes [10,11]. In the healthy brain, they maintain synaptic homeostasis, clear apoptotic cells and cellular debris, and secrete trophic factors that support neuronal and vascular function [12]. Early in cerebrovascular injury, microglia respond adaptively by phagocytosing debris, secreting neuroprotective factors, and participating in tissue remodeling. However, under conditions of chronic hypoperfusion and repeated ischemic stress, microglia shift toward a sustained pro-inflammatory phenotype, contributing to synaptic dysfunction, white matter damage, and progressive cognitive impairment [13,14].

Mechanistically, microglial responses in VaD are governed by interconnected signaling networks. Damage-associated molecular patterns (DAMPs) and extracellular ATP released from injured neural cells activate purinergic and Toll-like receptors (TLRs), which synergistically coordinate microglial chemotaxis and NLRP3 inflammasome assembly [15,16]. Meanwhile, the complement components (C1q/C3-CR3 axis) mediate synaptic tagging, driving aberrant synaptic pruning and circuit disruption [17,18]. Emerging evidence further implicates TREM2, a microglial metabolic sensor, and CSF1R, a regulator of survival and proliferation, in shaping disease-associated microglial (DAM) phenotypes [19,20]. Collectively, these pathways position microglia as central mediators of the NVU that underlies VaD pathogenesis. Given their dual and temporally dynamic roles, microglia serve as a critical link between cerebrovascular pathology and neurodegeneration. A comprehensive understanding of how microglial functions evolve across disease stages is essential for developing disease-modifying therapies that preserve their protective capacities while mitigating chronic inflammation.

This review synthesizes current findings on microglial involvement in VaD, focusing on temporal dynamics, key molecular mechanisms, and emerging therapeutic approaches for modulating microglial states to attenuate the trajectory of progressive cognitive decline. We propose that maladaptive microglial phenotypic shifts, driven by VaD-associated chronic neurovascular insults, constitute the critical mechanistic link between primary neurovascular unit disfunction and secondary neurocognitive impairment, providing a unifying framework to guide precision immunomodulatory therapeutics.

## 2. The Microglia–Neurovascular Interface in Health and Disease

NVU dysfunction is increasingly recognized as a central contributor to VaD pathogenesis. Within the NVU, microglia serve as dynamic regulators of neurovascular integrity, interacting with endothelial cells, pericytes, astrocytes, and neurons to maintain homeostasis. Rather than existing in a static inflammatory state, microglial responses are context-dependent and adapt to ongoing neurovascular stress and injury. Although the roles of microglia in NVU function and VaD have been described, their dynamic, stage-dependent evolution has not been formally conceptualized. Here, we propose a triphasic framework in which microglial responses progress through: (1) an early phase of adaptive surveillance and homeostatic support; (2) an intermediate phase characterized by metabolic stress and cellular reprogramming; and (3) a chronic phase marked by maladaptive neuroinflammation and progressive NVU dysfunction. While simplified, this framework provides a conceptual model linking initial cerebrovascular injury to cumulative NVU impairment and subsequent neurodegeneration, emphasizing the central role of microglial dynamics in VaD pathophysiology.

### 2.1. Microglia in the Neurovascular Unit: Guardians of Brain Health

Microglia are distributed throughout the brain parenchyma, where they interact closely with endothelial cells, pericytes, and astrocytes within the NVU [21]. In the healthy brain, highly ramified microglial processes continuously survey vessels and surrounding tissue, enabling the rapid detection of metabolic or hemodynamic disturbances. Microglia secrete trophic factors that support endothelial integrity, tight junction stability, and pericyte survival [22] while also removing cellular debris and metabolic waste within the perivascular space. These activities help sustain a local microenvironment that supports efficient nutrient exchange, neurovascular coupling, and overall neural function [23,24]. Through integrated roles in surveillance, trophic support, and waste clearance, microglia contribute to the dynamic regulation of NVU homeostasis and participate in early responses to neurovascular stress.

### 2.2. Functional Plasticity of Microglial Responses

Following acute cerebral hypoperfusion or small vessel insults, microglia rapidly respond to sites of vascular and parenchymal injury through process extension and chemotaxis [12,25]. Activated microglia phagocytose apoptotic cells, myelin debris, and extracellular protein aggregates, preventing secondary necrosis and limiting the propagation of inflammatory signals [26]. Concurrently, they release neurotrophic and anti-inflammatory mediators, including BDNF, GDNF, NGF, IL-10, and TGF-β, which support neuronal survival, enhance synaptic plasticity, and stabilize the local microenvironment [27,28,29]. These early responses help maintain NVU integrity through coordinated interactions with endothelial cells and astrocytes, thereby preserving microcirculatory function and supporting tissue repair [6,30].

Chronic cerebral hypoperfusion and recurrent vascular insults drive metabolic and phenotypic reprogramming of microglia [31]. Under these conditions, microglia adopt a pro-inflammatory profile characterized by excessive cytokine release, oxidative and nitrosative stress, impaired debris clearance, and pathological interactions with NVU components [32,33]. This chronic activation amplifies neuronal vulnerability, accelerates white matter damage, and contributes to progressive NVU dysfunction [15,34,35]. The transition from adaptive defense to maladaptive inflammation therefore represents a critical inflection point in the progression of vascular dementia.

## 3. Key Signaling Pathways Governing Microglial Dynamics and Reprogramming

**Figure 1:**
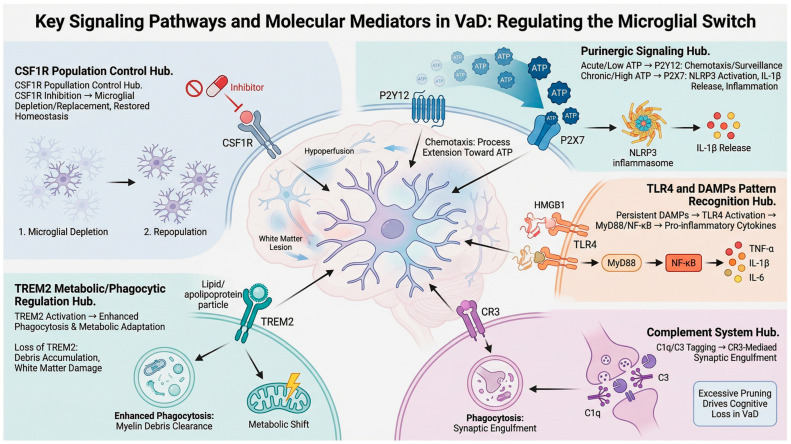
Molecular pathways regulating the microglial phenotypic switch in vascular dementia. This schematic integrates major signaling pathways governing microglial dysfunction in VaD. CSF1R signaling supports microglial survival and enables depletion–repopulation to restore homeostasis. Purinergic signaling is concentration-dependent: transient ATP elevations promote P2Y12-mediated chemotaxis and surveillance, whereas sustained ATP elevations activate P2X7-dependent inflammasome activation. TREM2 regulates lipid sensing, phagocytosis, and metabolic adaptation; its loss leads to debris accumulation and white matter damage. TLR4/DAMP signaling activates MyD88/NF-κB signaling, inducing pro-inflammatory cytokine production. Complement signaling mediates C1q/C3 tagging of synapses followed by CR3-dependent microglial phagocytosis, contributing to synaptic loss.

**Figure 2:**
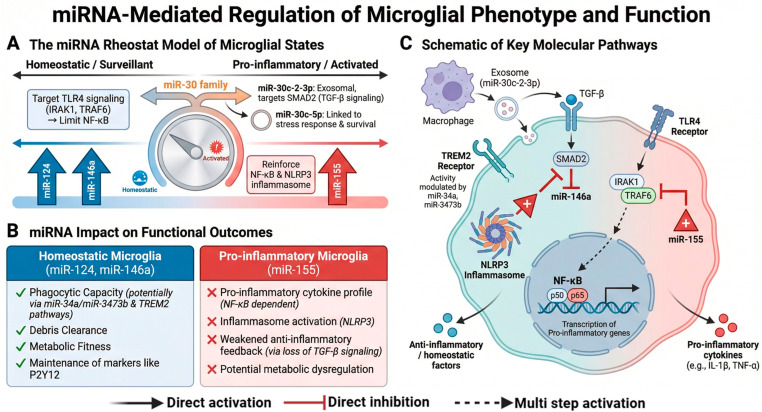
miRNA-Mediated Regulation of Microglial Phenotype. (**A**) The miRNA rheostat model: miR-124/146a constrain microglial activation by targeting TLR4 (IRAK1/TRAF6), limiting NF-κB; miR-155 amplifies inflammation via NF-κB/NLRP3; miR-30 family exerts context-dependent effects (exosomal miR-30c-2-3p targets SMAD2, promoting inflammation). (**B**) Functional outcomes: homeostatic miRNAs support phagocytosis (miR-34a/3473b-TREM2), debris clearance, and metabolic fitness; miR-155 drives cytokines, inflammasome activation, and metabolic dysregulation. (**C**) Schematic illustrating these integrated pathways, where peripheral macrophage-derived exosomal miR-30c-2-3p inhibits SMAD2, while miR-146a and miR-155 modulate TLR4/NF-κB signaling.

The transition of microglia from homeostatic sentinels to chronically inflammatory effectors in VaD pathogenesis is orchestrated by interconnected molecular signaling networks that regulate chemotaxis, phagocytosis, metabolic fitness, and cytokine production [14]. Rather than a simple binary switch, microglial activation represents a dynamic continuum of functional states, continuously reshaped by persistent cerebrovascular insults and reciprocal signaling within the neurovascular unit. Increasing evidence indicates that these pathways not only drive dysfunction but also remain pharmacologically targetable, offering opportunities to reprogram microglia toward reparative phenotypes [36]. Here, we review key regulatory systems including purinergic receptors, pattern-recognition pathways, complement signaling, and metabolic and survival regulators (Figure 1), as well as post-transcriptional regulation by miRNAs (Figure 2).

### 3.1. Purinergic Signaling: From Directed Surveillance to Inflammasome Activation

Extracellular nucleotides act as early “danger signals” released after ischemic or hypoperfusion injury. ATP and its metabolic ADP rapidly activate purinergic receptors on microglia, guiding chemotactic migration toward sites of tissue damage. Under physiological or acute conditions, the P2Y12 receptor, a hallmarker of homeostatic microglia, mediates process extension and targeted debris clearance [16,37]. This hyperacute response enables the rapid containment of injury and supports tissue repair.

However, sustained extracellular ATP accumulation during chronic hypoperfusion leads to the downregulation of P2Y12 and preferentially activates P2X7, a high-threshold ATP-gated channel that mediates pro-inflammatory signaling. Prolonged P2X7 stimulation promotes assembly of the NLRP3 inflammasome, caspase-1 activation, and release of IL-1β and other pro-inflammatory cytokines, thereby amplifying neurotoxicity and blood–brain barrier disruption [15,38,39]. Accordingly, the P2X7/P2Y12 ratio represents an emerging molecular signature of microglial functional state, providing a sensitive index of the transition from homeostatic surveillance to a pro-inflammatory, neurotoxic phenotype [40,41]. Collectively, purinergic signaling functions as a molecular switch that governs microglial transition from protective chemotaxis to chronic inflammatory activation.

### 3.2. Pattern Recognition Pathways: TLR4 and Inflammasome Amplification

Microglia detect endogenous injury through a sophisticated repertoire of pattern-recognition receptors (PRRs). Among these, Toll-like receptor 4 (TLR4) serves as a primary sensor for sterile neuroinflammation. DAMPs released from ischemic or necrotic cells, most notably HMGB1 and heat-shock proteins, activate TLR4 and trigger MyD88-dependent NF-κB signaling. This pathway induces the transcription of pro-inflammatory mediators, including TNF-α and pro-IL-1β [42,43,44]. Persistent cerebrovascular injury leads to continuous DAMP exposure, maintaining microglia in a reactive state that promotes endothelial dysfunction and blood–brain barrier breakdown. Critically, the convergence of this TLR4-mediated “priming” with P2X7-dependent activation of the NLRP3 inflammasome amplifies cytokine maturation and establishes a self-sustaining inflammatory loop that drives progressive neurovascular damage [45].

### 3.3. Complement Signaling: Aberrant Synaptic Elimination

The complement cascade links inflammation to synaptic and myelin disruption. During development, C1q and C3 tag weak synapses for pruning, enabling circuit refinement [46]. In vascular dementia, chronic ischemia and oxidative stress reactivate this program, while BBB disruption enhances complementary and local deposition within the NVU. Within the NVU, microglia produce C1q, whereas C3 is derived from both reactive astrocytes and microglia. Together, C1q and C3 deposit on vulnerable synapses, which are recognized by microglial complement receptor 3 (CR3) and eliminated via phagocytosis [17,18,46]. Beyond synapses, complement activation contributes to white matter injury through microglia-mediated myelin phagocytosis and oligodendrocyte damage. This aberrant pruning disrupts hippocampal and cortical networks essential for cognition and reinforce pro-inflammatory microglial states under chronic cerebral hypoperfusion [47]. Collectively, complement-mediated dysregulation represents a key mechanism linking chronic neurovascular injury to progressive cognitive decline in vascular dementia.

### 3.4. Metabolic and Reparative Regulation: TREM2 Signaling

Beyond inflammatory sensing, microglial function is closely linked to metabolic fitness. TREM2 serves as a lipid-sensing receptor that promotes microglial survival, phagocytosis, and metabolic adaptation. Activation of TREM2 facilitates the clearance of apoptotic cells and myelin debris and supports a reparative, disease-associated microglial phenotype characterized by enhanced lipid metabolism and tissue remodeling [19,48,49]. Disruption of TREM2 signaling impairs debris removal, promotes chronic inflammation, and exacerbates neuronal injury. Consequently, genetic or functional deficits in this pathway may compromise the transition toward protective states, influencing whether microglia contribute to neurovascular recovery or progressive degeneration [50]. Although TREM2 is well-established as a regulator of microglial metabolism and phagocytic function in Alzheimer’s disease and other neurodegenerative disorders, its specific role in vascular dementia remains poorly defined. Direct experimental evidence in VaD-specific models is currently limited.

### 3.5. Population Dynamics and Reprogramming: CSF1R Signaling

Microglial identity and survival depend on CSF1R signaling. Pharmacological inhibition of CSF1R can transiently deplete microglia, followed by repopulation from residual, progenitor-like cells that adopt less inflammatory and more homeostatic phenotypes [20,51]. In experimental models, this “microglial resetting” attenuates pro-inflammatory cytokine profiles, enhances debris clearance, and improves white matter integrity [20,52]. Collectively, these findings demonstrate the remarkable phenotypic plasticity of microglia and suggest that targeted therapeutic reprogramming, rather than broad immunosuppression, represents a promising strategy to restore homeostatic balance within the NVU in VaD and related neurovascular disorders.

### 3.6. Post-Transcriptional Regulation of Microglial Phenotypes: Emerging Roles of MicroRNAs

Beyond receptor-mediated and metabolic regulation, microRNAs (miRNAs) have emerged as key modulators of microglial plasticity, acting as molecular “brakes” or “amplifiers” of neuroinflammatory responses [53] (Figure 2). Under physiological conditions, brain-enriched miR-124 and the negative feedback regulator miR-146a help maintain microglial homeostasis by targeting components of the TLR4 signaling cascade, including IRAK1 and TRAF6, thereby limiting NF-κB-dependent cytokine production [54,55]. Conversely, under pathological conditions, microglial miRNA expression can shift toward a pro-inflammatory profile. For example, miR-155 is often upregulated in activated microglia, where it can enhance NF-κB-mediated cytokine production and suppresses negative regulators such as SOCS1 and SHIP1 [56]. IFN-γ may further promote miR-155 expression, creating a feedback loop that helps maintain microglial activation [57,58]. Together, these observations suggest that miR-155 acts as a key molecular hub shaping pro-inflammatory microglial states under pathological conditions.

Members of the miR-30 family provide an additional layer of context-dependent regulation of microglial functional phenotypes. For example, miR-30a is upregulated in activated microglia during the chronic phase of EAE, where it promotes a pro-inflammatory phenotype by increasing IL-1β and iNOS (inducible nitric oxide synthase) expression while suppressing anti-inflammatory markers Ym-1 and CD206 [59]. miR-30c-5p is downregulated in microglia during cryptococcal meningitis, leading to the activation of the eIF2α/ATF4 pathway; its restoration suppresses the inflammatory response, apoptosis, and autophagic activity [60]. Additionally, exosomal miR-30c-2-3p has been shown to exacerbate microglial neuroinflammation during cerebral ischemia by targeting the TGF-β/SMAD2 pathway [61]. Collectively, the miR-30 family exerts highly context-specific effects on microglial functional phenotypes, promoting either neuroprotective or pro-inflammatory responses depending on the context of disease.

Beyond inflammatory signaling, emerging evidence suggests that certain microRNAs may also influence microglial metabolic and homeostatic functions. For example, miR-34a and miR-3473b have been implicated in modulating TREM2 signaling and microglial activation states [62,63], although the precise regulatory mechanisms in microglia remain incompletely defined.

### 3.7. Integrated Perspective

Collectively, these signaling networks constitute an interconnected regulatory system that governs microglial state transitions. Early purinergic and TREM2 signaling support microglial surveillance and reparative functions, whereas sustained extracellular ATP exposure, TLR4 activation, inflammasome assembly, and complement reactivation promote inflammatory and synaptotoxic phenotypes. Importantly, these pathways appear at least partially reversible, supporting the concept that microglial dysfunction reflects maladaptive reprogramming rather than irreversible cellular damage. In this context, microRNAs emerge as higher-order post-transcriptional regulators that fine-tune these interconnected pathways, thereby coordinating the balance between protective and detrimental microglial responses as disease progresses.

Deciphering the spatiotemporal interaction of these molecular circuits provides a mechanistic framework for developing precision therapeutics that selectively attenuate maladaptive signaling while preserving essential homeostatic functions. While many of these mechanisms likely contribute to VaD pathogenesis, most current insights are extrapolated from related neurodegenerative or cerebrovascular disorders. This highlights that our understanding of microglial reprogramming in VaD remains incomplete, underscoring the need for further validation in dedicated preclinical and human studies.

## 4. Dual Role of Microglia in Vascular Dementia: From Neuroprotection to Neurotoxicity

**Figure 3:**
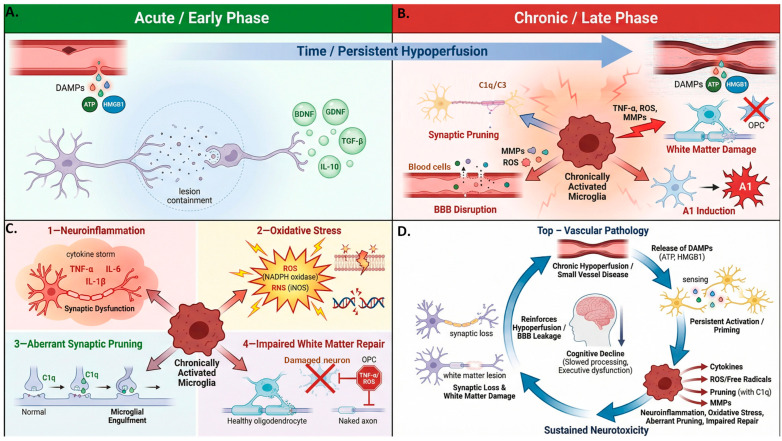
Microglial Dynamics in Vascular Dementia. This multi-panel schematic illustrates the temporal evolution of microglial responses following cerebrovascular injury. (**A**) Acute vascular injury (leaky vessel releasing DAMPs) triggers adaptive microglial responses: ramified microglia migrate, phagocytose debris, and release neurotrophic factors (BDNF, GDNF, TGF-β, IL-10) while containing lesion spread. (**B**) Conversely, chronic hypoperfusion (narrowed vessel with persistent DAMPs) drives maladaptive transformation into amoeboid, chronically activated microglia. (**C**) These cells become neurotoxic hubs, radiating four pathological mechanisms: neuroinflammation (cytokines), oxidative stress (ROS/RNS), aberrant complement-mediated synaptic pruning (C1q/C3), and impaired white matter repair (oligodendrocyte toxicity, OPC blockade). (**D**) A self-reinforcing vicious cycle emerges vascular pathology → DAMP release → microglial priming → sustained neurotoxicity → synaptic/white matter damage → further vascular compromise, ultimately culminating in progressive cognitive decline.

Microglia exhibit stage-dependent and context-specific responses in VaD. Following cerebral hypoperfusion or ischemic injury, they initially adopt neuroprotective functions that support debris clearance and neurovascular homeostasis. However, persistent vascular dysfunction and unresolved inflammatory signals drive a shift toward chronic pro-inflammatory activation. This transition from adaptive to maladaptive states contributes to synaptic loss, white matter injury, and progressive cognitive decline in VaD [14] (Figure 3).

### 4.1. Early Adaptive Responses: Stabilizing the Injured Brain

Immediately following acute ischemic or hypoperfusion events, microglia adopt a reparative phenotype aimed at restoring tissue homeostasis. They rapidly migrate to sites of injury, where they phagocytose apoptotic neurons, myelin debris, and other damage-associated material, thereby limiting secondary inflammation and facilitating tissue repair [25]. Microglia also release neurotrophic and anti-inflammatory mediators, including BDNF, GDNF, NGF, IL-10, and TGF-β, which support neuronal survival, synaptic plasticity, and inflammatory resolution [64]. Spatially, microglia also contribute to lesion containment by forming cellular and molecular barriers around damaged regions [36]. Collectively, these early responses help confine injury, preserve surrounding neural circuits, and promote functional recovery.

### 4.2. Chronic Maladaptation: Persistent Activation and Neurotoxicity

Following the early reparative phase, microglia in VaD can adopt maladaptive phenotypes. Persistent activation drives chronic inflammation, oxidative stress, synaptic loss, and impaired white matter repair, setting the stage for progressive neural dysfunction.

*Sustained Neuroinflammation.* In experimental models of CCH that recapitulate key pathological features of VaD, chronically activated microglia exhibit a sustained production of pro-inflammatory cytokines, including TNF-α, IL-1β, and IL-6. This persistent inflammatory signaling has been associated with progressive neural dysfunction [4,13]. Prolonged exposure to these mediators may establish self-perpetuating inflammatory feedback loops that disrupt neuronal–glial communication, impair synaptic plasticity, and increase susceptibility to excitotoxic stress [64]. Although elevated circulating proinflammatory markers correlate with cognitive impairment in patients with VaD [65,66], it remains unclear whether these systemic signals accurately reflect microglial activity within the brain or instead represent secondary consequences of broader peripheral inflammation. Furthermore, the precise mechanisms governing the transition of microglia from an early compensatory response to a chronic, self-sustaining inflammatory state during VaD progression remain incompletely defined.

*Oxidative and Nitrosative Stress.* Persistent microglial activation promotes excessive generation of reactive oxygen species (ROS) and reactive nitrogen species (RNS), primarily through the NADPH oxidase (NOX) and iNOS pathways [13,67]. While these molecules function as signaling mediators at physiological levels, their chronic overproduction can induce oxidative damage to lipids, proteins, and DNA across neurons, oligodendrocytes, and endothelial cells. This cumulative oxidative burden contributes to NVU dysfunction, manifesting as cerebrovascular impairment and demyelination that may further exacerbate hypoperfusion and chronic injury in VaD. Despite these associations, the precise spatiotemporal dynamics and regulatory mechanisms linking microglial-derived ROS/RNS to neurovascular dysfunction in VaD remain incompletely understood. Clarifying how oxidative and nitrosative stress interact with microglial activation and NVU integrity will be important for determining whether these pathways represent viable therapeutic targets in vascular cognitive impairment.

*Aberrant Synaptic Pruning.* Synaptic remodeling is a physiological process essential for circuit refinement and adaptive neural responses, however, microglia may exhibit heightened phagocytic activity, potentially leading to aberrant synaptic engulfment and structural synapse loss [68,69]. Inflammatory and oxidative signals can enhance microglial recognition and the removal of vulnerable synapses, thereby disrupting neuronal connectivity and network function [46,47]. Notably, complement-dependent synaptic pruning pathways (e.g., C1q, C3, and CR3), which normally function during development, may be aberrantly reactivated in the adult brain. Evidence from multiple neurodegenerative disorders suggests that dysregulated microglia-mediated synaptic removal contributes to cognitive impairment [46,70]. However, it remains unclear whether similar complement-dependent mechanisms are directly triggered by the chronic hypoperfusion and ischemic stress characteristic of VaD.

*Impaired White Matter Repair*. Persistent microglial activation disrupts white matter repair in vascular dementia by promoting oligodendrocyte injury and creating an inflammatory milieu that limits oligodendrocyte precursor cell (OPC) differentiation and remyelination. This represents a key mechanistic distinction from gray matter synaptic pruning: while both processes involve microglial phagocytosis, white matter pathology is dominated by myelin debris clearance and impaired OPC support.

*Myelin phagocytosis and debris clearance*. In CCH models, microglial activation occurs early and spatially overlaps with demyelination and oligodendrocyte loss in vulnerable white matter tracts, including the corpus callosum and periventricular regions [71,72]. Activated microglia release inflammatory mediators, reactive oxygen species, and matrix metalloproteinases that can damage oligodendrocytes and myelin. Although acute myelin clearance is initially protective, chronic lipid overload can drive microglial foam cell-like transformation, inflammasome activation, and impaired phagocytic efficiency, creating a self-perpetuating cycle of failed debris clearance.

*OPC interactions and remyelination failure*. Under physiological conditions, microglia support OPC survival and differentiation via trophic factors such as IGF-1, PDGF-AA, and TGF-β. In chronic hypoperfusion, however, microglia adopt a pro-inflammatory phenotype characterized by TNF-α, IL-1β, IFN-γ, and oxidative stress, which collectively inhibits OPC maturation and leads to OPC accumulation without effective remyelination. In addition, complement C3/C3aR signaling contributes to microglia-mediated white matter injury in CCH models [73].

Clinical evidence supports these experimental findings. Postmortem studies of vascular dementia and post-stroke dementia reveal increased densities of CD68+ microglia within deep white matter lesions, often colocalizing with degraded myelin basic protein [74,75]. Neuroimaging studies further link reduced white matter integrity to impaired processing speed and executive dysfunction [76]. Together, these findings support a mechanistic role for chronic microglial activation in impairing white matter repair and driving lesion progression in vascular dementia.

## 5. Therapeutic Implications and Future Directions

**Figure 4:**
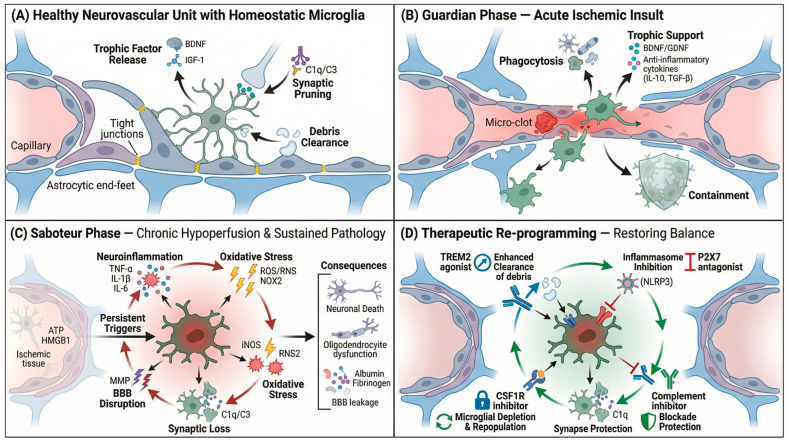
Microglial States across the Vascular Dementia Spectrum. This four-panel schematic depicts microglial evolution within the neurovascular unit. (**A**) Healthy homeostasis: ramified microglia release trophic factors (BDNF, IGF-1), clear micro-clots, and maintain surveillance. (**B**) Guardian phase (acute ischemia): microglia adopt protective functions—phagocytosing debris (C1q/C3) and mounting controlled neuroinflammation (TNF-α, IL-1β, IL-6) with transient oxidative stress. Persistent triggers (ATP, HMGB1) accumulate. (**C**) Saboteur phase (chronic hypoperfusion): microglia transform into persistently activated, neurotoxic cells driving sustained inflammation, oxidative stress (NOX2, iNOS), BBB disruption, and complement-mediated damage—underpinning synaptic loss and white matter degeneration. (**D**) Therapeutic reprogramming: pharmacological strategies (TREM2 agonism, CSF1R inhibition, complement/MMP/iNOS blockade) restore homeostasis, promoting synapse protection and reducing neurotoxicity.

In VaD, microglial dysfunction arises primarily from maladaptive and persistent activation rather than activation itself. Because microglia are essential for debris clearance, synaptic maintenance, and vascular support, indiscriminate immunosuppression may impair protective functions and hinder tissue repair. Consequently, emerging therapeutic strategies are shifting from broad anti-inflammatory approaches toward the selective modulation of microglial states aimed at restoring homeostatic functions while limiting chronic neurotoxicity (Figure 4).

### 5.1. Reprogramming Rather than Suppressing Microglia

Early attempts to attenuate neuroinflammation using repurposed anti-inflammatory agents, such as NSAIDs and minocycline, have shown limited and inconsistent clinical benefit. These findings highlight a central challenge: non-specific immunosuppression fails to distinguish protective from detrimental microglial functions. Because microglia exhibit stage-dependent roles following neurovascular injury, therapeutic efficacy is critically influenced by the timing of intervention. Early-phase modulation may preserve homeostatic and repair-supportive activities, whereas later-state interventions must target chronically activated and maladaptive microglial states [77].

Accordingly, emerging strategies aim to recalibrate rather than suppress microglial activity. CSF1R signaling is essential for microglial survival and maintenance; pharmacological inhibition of CSF1R induces near-complete depletion of microglia, followed by rapid repopulation after drug withdrawal. Repopulated microglia often exhibit transcriptional profiles closer to homeostatic states and may partially reverse disease-associated signatures, although they can retain distinct molecular characteristics compared to the original populations [51]. In preclinical models of chronic cerebral hypoperfusion and small vessel disease, microglia depletion–repopulation strategies suppress chronic neuroinflammation, reduce white matter injury, and improve cognitive performance [78,79]. It should be noted that this approach is not selective for activated microglia and may also affect other CNS-associated macrophages, including perivascular macrophages. In addition, therapeutic efficacy appears highly stage-dependent, with greater benefit in early-to-intermediate disease stages [80,81]. Together, these findings support microglial reprogramming aimed at restoring functional homeostasis as a mechanistically grounded therapeutic direction in vascular dementia.

Beyond population-level resetting, receptor-specific reprogramming strategies are also being explored. For example, activation of TREM2 enhances microglial phagocytic capacity, promotes debris clearance, and limits excessive pro-inflammatory cytokine production in models of neurodegenerative diseases, with reported improvements in cognitive outcomes [82,83]. However, translation remains challenging, as most existing agonistic tools are antibody-based and exhibit limited blood–brain barrier penetration.

Other approaches aim to selectively inhibit maladaptive inflammatory signaling while preserving basal immune surveillance. For example, studies in Alzheimer’s disease models demonstrate that P2X7 receptor activation promotes NLRP3 inflammasome assembly and pro-inflammatory cytokine release in microglia, whereas pharmacological inhibition of this pathway reduces chronic neuroinflammation and improves cognitive outcomes [84,85]. Although direct evidence for a microglial P2X7–NLRP3 axis in vascular dementia remains limited, indirect data suggest its potential involvement in vascular cognitive impairment [86,87,88].

Collectively, these strategies reflect a shift from broad anti-inflammatory suppression toward selective reprogramming of microglial states and signaling pathways to restore immune and metabolic balance within the neurovascular unit. Future studies should integrate microglial function with neurovascular and systemic modulators of disease progression to better define their contributions to cognitive decline.

### 5.2. Translational Challenges and Future Directions

While reprogramming rather than suppressing microglia represents a promising conceptual framework, translating this principle into clinical interventions presents substantial challenges.

First, many candidate interventions raise important safety and feasibility concerns. For example, pharmacological inhibition of CSF1R, widely used in experimental models to deplete microglia, also affects peripheral macrophage populations because CSF1R signaling is shared across myeloid lineages. Clinical use of CSF1R inhibitors in oncology has been associated with systemic toxicities, including hepatotoxicity, highlighting the difficulty of selectively targeting microglia without disrupting peripheral immune homeostasis [89,90]. Therefore, future therapeutic strategies must prioritize cell-specific modulation within the CNS while minimizing systemic immune perturbation.

Second, therapeutic efficacy is highly dependent on disease stage, reflecting the temporal evolution of microglial phenotypes across the diverse spectrum of VaD, including CCH and cSVD. During early phases of neurovascular injury, microglia contribute to NVU homeostasis through debris clearance and support of neurovascular coupling, and broad inhibition at this stage may impair endogenous repair mechanisms. In contrast, during chronic disease progression, microglia often adopt a persistent pro-inflammatory phenotype characterized by the secretion of matrix-degrading enzymes and other neurotoxic mediators that contribute to NVU dysfunction and white matter injury. Accordingly, we propose that successful therapeutic strategies will require the stage-specific modulation of microglial states rather than non-selective, uniform suppression.

Third, most microglia-targeting interventions in vascular dementia remain at the preclinical stage. While CCH and cSVD models suggest that shifting microglia toward a reparative phenotype can reduce white matter damage [73,91], robust clinical validation in humans is still lacking. Bridging this gap will require more pathophysiologically relevant models, including aged animals and human iPSC-derived NVU systems, to better capture multicellular interactions among microglia, neurons, and the cerebral vasculature.

Ultimately, moving from blanket inhibition toward the restoration of microglial homeostatic regulation represents a more viable therapeutic direction in vascular dementia. Such approaches aim to attenuate chronic neurotoxicity and maladaptive synaptic and white matter injury repair while preserving repair-supportive functions.

### 5.3. Biomarkers and Patient Stratification

Reliable biomarkers capable of monitoring microglial functional states are urgently needed to enable clinically feasible microglia-targeted therapies. Current TSPO-PET (translocator protein-positron emission tomography) imaging, which detects the 18 kDa translocator protein expressed primarily in activated microglia but also in astrocytes and endothelial cells, has been widely used to assess neuroinflammation in vivo but has significant limitations. TSPO ligands do not reliably distinguish microglial functional phenotypes (e.g., protective vs. detrimental), and TSPO signals largely reflect the overall inflammatory cell density rather than distinct microglial states. In addition, inter-individual variability caused by genetic polymorphisms and non-microglial TSPO expression further complicates interpretation across patients and disease contexts [92,93]. Consequently, the development of next-generation radiotracers, blood-based biomarkers, and transcriptomic or extracellular vesicle-derived signatures capable of resolving microglial functional states will be essential for patient stratification and for defining optimal therapeutic windows in vascular dementia.

### 5.4. Molecular, Biological, and Systemic Modulators

Microglial phenotypic diversity is governed by interconnected signaling pathways that offer opportunities for targeted modulation rather than broad immunosuppression. Key regulators include purinergic receptors, Toll-like receptor–mediated signaling, inflammasome cascades, complement-mediated synaptic pruning, and metabolic checkpoints such as TREM2 and CSF1R. In addition, post-transcriptional mechanisms, particularly microRNAs (miR-124, miR-146a, miR-155, and members of the miR-30 family), have emerged as important regulators that fine-tune inflammatory and reparative microglial states. These miRNAs are detectable in cerebrospinal fluid, plasma, and extracellular vesicles, providing potential biomarkers of microglial activation as well as candidate therapeutic tools.

However, several translational barriers remain, including challenges in achieving cell-specific delivery across the blood–brain barrier, potential off-target effects in peripheral immune cells, and the difficulty of monitoring microglial functional states in patients. Overcoming these obstacles will be critical for translating microglial reprogramming strategies from experimental systems into clinically viable therapies.

Biological variability represents another important consideration for therapeutic translation. Sex-dependent differences in microglial density, transcriptional programs, and inflammatory responses have been documented and may influence both disease progression and treatment efficacy [94,95]. Accordingly, future experimental studies and clinical trials should incorporate sex as a biological variable when evaluating microglial-targeted therapies.

In addition, increasing attention is being directed toward systemic modulators of neuroinflammation, particularly the microbiota–gut–brain axis [96]. Microbial metabolites and immune signals derived from the gut microbiome can influence microglial maturation, immune tone, and inflammatory responsiveness. These findings suggest that dietary, metabolic, or microbiome-based interventions may complement pharmacological strategies, particularly in patients with vascular risk factors. Nevertheless, the clinical feasibility and reproducibility of such strategies remain uncertain, and further mechanistic and translational studies will be required to determine their therapeutic potential in vascular dementia.

## 6. Conclusions

Vascular dementia emerges from the interplay of chronic cerebrovascular dysfunction and progressive neurodegeneration, with microglia serving as central mediators of this crosstalk. While initially protective, maintaining homeostasis and clearing debris, persistent hypoperfusion drives toward maladaptive activation, fueling chronic inflammation and synaptic loss. Recognizing microglia as dynamic regulators rather than static effectors necessitates a shift in therapeutic strategy: precision modulation that restores balance and preserves reparative capacity is far more promising than indiscriminate immunosuppression. Future research should prioritize integrating single-cell transcriptomics, spatial profiling, and longitudinal imaging to delineate stage-specific microglial states amenable to precision reprogramming. Ultimately, the goal is to shift the microglial profile from a degenerative to a reparative role by targeting specific inflammatory pathways while sparing homeostatic functions. Achieving this will require the identification of molecular and systemic biomarkers to define patient-specific therapeutic windows, providing a rational framework to finally slow or prevent the progression of VaD.

## Data Availability

No new data were created or analyzed in this study. Data sharing is not applicable to this article.
